# Amino Acids Are an Ineffective Fertilizer for *Dunaliella* spp. Growth

**DOI:** 10.3389/fpls.2017.00847

**Published:** 2017-05-26

**Authors:** Colin A. Murphree, Jacob T. Dums, Siddharth K. Jain, Chengsong Zhao, Danielle Y. Young, Nicole Khoshnoodi, Andrey Tikunov, Jeffrey Macdonald, Guillaume Pilot, Heike Sederoff

**Affiliations:** ^1^Department of Plant and Microbial Biology, North Carolina State University, RaleighNC, United States; ^2^Department of Plant Pathology, Physiology, and Weed Science, Virginia Polytechnic Institute and State University, BlacksburgVA, United States; ^3^Research Triangle High School, Durham CountyNC, United States; ^4^Department of Biomedical Engineering, University of North Carolina School of Medicine, Chapel HillNC, United States

**Keywords:** Dunaliella, nitrogen recycling, lipids, biofuel, amino acids, sustainability

## Abstract

Autotrophic microalgae are a promising bioproducts platform. However, the fundamental requirements these organisms have for nitrogen fertilizer severely limit the impact and scale of their cultivation. As an alternative to inorganic fertilizers, we investigated the possibility of using amino acids from deconstructed biomass as a nitrogen source in the genus *Dunaliella.* We found that only four amino acids (glutamine, histidine, cysteine, and tryptophan) rescue *Dunaliella* spp. growth in nitrogen depleted media, and that supplementation of these amino acids altered the metabolic profile of *Dunaliella* cells. Our investigations revealed that histidine is transported across the cell membrane, and that glutamine and cysteine are not transported. Rather, glutamine, cysteine, and tryptophan are degraded in solution by a set of oxidative chemical reactions, releasing ammonium that in turn supports growth. Utilization of biomass-derived amino acids is therefore not a suitable option unless additional amino acid nitrogen uptake is enabled through genetic modifications of these algae.

## Introduction

Autotroph algae have gained attention in recent years because they are potentially a valuable bioproduct feedstock. These organisms could produce large quantities of high value proteins, chemicals, or combustible hydrocarbons on a relatively small acreage, and may operate with less environmental impact than conventional agriculture or fossil fuels (Wijffels and Barbosa, 2010; Georgianna and Mayfield, 2012; Rasala and Mayfield, 2015). The theoretical energy yield for biofuel production from algae per unit area is estimated to be between 30- and 300-fold higher than any available crop system (Sheehan et al., 1998; Packer, 2009), and these organisms may eventually be productive enough to satisfy a large proportion of fuel consumption using non-arable land (US Department of Energy, 2010; Moody et al., 2014). In addition, some fuel producing microalgae thrive in marine environments, and therefore their production could be supported entirely using non-potable water sources such as ocean water. However, the viability of growing algae at scale for any purpose is limited by the requirements these organisms have for nitrogen and phosphorous fertilizers. The demand for these fertilizers is such that if only 10% of transportation fuels needed in the US for 2010 were replaced by algal biodiesel, it would require an amount of nitrogen fertilizer equivalent to 175% of total annual US production (Grobbelaar, 2004; Chisti, 2013). This is considerable, as the production of synthetic N-fertilizer via the Haber-Bosch process is reliant on energy derived from fossil fuels. Furthermore, the use of fertilizer in agriculture effectively creates nitrogen pollution in the form of nitrous oxides, a greenhouse gas, which is thought to offset gains in the global warming potential of biofuels (Crutzen et al., 2008; Erisman et al., 2008; Galloway et al., 2008; Bobbink et al., 2010). There is therefore a need to address the impacts of nitrogen fertilizer use within the bioeconomy.

One solution is to reduce both fertilizer inputs and nitrogen waste by creating an effective nitrogen recycling scheme. Many groups have proposed using thermochemical conversion and anaerobic digestion of biomass as potential sources of recycled nitrogen (Minowa et al., 1995; Chisti, 2008; Sialve et al., 2009; Jena et al., 2011; Biller et al., 2012; Huo et al., 2012; Garcia Alba et al., 2013; López Barreiro et al., 2013a). These degradative approaches have the benefit that phosphorous and nitrogen can be resupplied in an inorganic form, and theoretically enable the possibility of growing multiple culture generations from a single fertilizer input. However, the feasibility of these approaches remains questionable because of the high nitrogen content of biomass and the energetic costs of concentrating algae for these applications (Mata-Alvarez et al., 2000; Chen et al., 2008; Lam and Lee, 2012; López Barreiro et al., 2013b; Ward et al., 2014). Both approaches result in the production of ammonium as the most abundant form of recycled nitrogen (Tsukahara et al., 2001; Sialve et al., 2009). Consequently, the pH of cultivation medium and ammonium concentration must be tightly controlled in any resulting algal growth system to prevent toxicity resulting from electrochemical gradient decoupling (Azov and Goldman, 1982). Furthermore, these approaches may be incompatible with the use of marine and saline algae feedstocks, as the salt content of this biomass is corrosive or inhibitory to thermochemical and biological degradation schemes (McCarty, 1964; Parkin and Owen, 1986; Chen et al., 2008; Vergara-Fernández et al., 2008; Ward et al., 2014).

**FIGURE 1:**
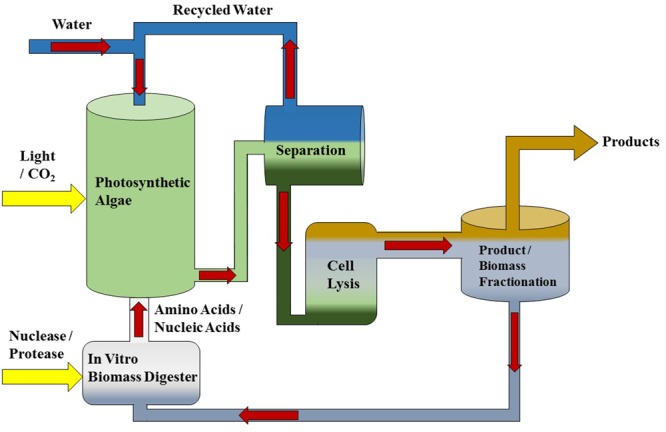
**Diagram of a microalgae production system featuring *in vitro* biomass recycling.** This production system is intended to work with any photosynthetic microalgae platform such as *Dunaliella* that produces extractable lipids or high value co-products. *In vitro* nutrient recycling is performed in a separate bioreactor to generate N and P organic monomers from algal biomass that are intended as a substitute for fertilizer additions.

An alternative scheme would be to recycle the nitrogen-rich biomass of algae in an organic form. The proposed strategy is to use an *in vitro* process to degrade algal biomass (proteins and nucleic acids) after extraction of non-polar lipid products into nitrogen-containing organic monomers (amino acids, nucleotides), and then to replace inorganic nitrogen fertilizer in algae production with those monomers (**Figure 1**). Specifically, protease and nuclease enzymes would be applied to algal biomass in a bioreactor to generate amino and nucleic acids in the same manner as amylase is added to starch to generate the sugars used in the industrial production of ethanol. The enzymatically treated biomass slurry would then be introduced into the algal cultivation medium in lieu of inorganic nitrogen and phosphorous fertilizer. Importantly, this strategy relies on the capacity of a species of alga to use organic nitrogen and phosphorous containing compounds as a fertilizer source. It is also imperative that utilization of these organic nitrogen and phosphorous monomers maintains the productivity of a desired product, such as triacylglycerols (TAGs).

There is reason to suspect that biofuel-producing autotrophic algae would be able to use amino acids, as marine and freshwater autotrophic algae have demonstrated the ability to grow on amino acid-supplemented media (Cain, 1965; Wheeler et al., 1974; Palenik and Morel, 1990; Zhang et al., 2015). Autotrophic algae have at least two mechanisms by which the nitrogen from amino acids could be acquired. Like plants (Fischer et al., 1998; Tegeder, 2012; Tegeder and Ward, 2014), algae possess channels or transporters that could facilitate movement of amino acids into the cell (Kirk and Kirk, 1978a,b; Sauer, 1984; Cho and Le Gal, 1985). In addition, it has been shown that algae possess extracellular enzymes that deaminate amino moieties, releasing ammonium that can be transported across the cell membrane and subsequently assimilated via the GS-GOGAT cycle (Munoz-Blanco et al., 1990; Palenik and Morel, 1990, 1991; Piedras et al., 1992; Vallon et al., 1993) into biomass. Some amino acids are unstable when present in aqueous solution and when exposed to light, forming NH_4_^+^, urea, and other amino acids over time (Asquith and Hirst, 1969; Tomita et al., 1969; Abraham and Podell, 1981; Gracanin et al., 2009; Pattison et al., 2012).

The viability and effects of feeding amino acids as a nitrogen source was investigated using the genus *Dunaliella*. *Dunaliella* spp. are motile, unicellular, autotrophic, halophitic algae that are often isolated from extreme saline environments (Borowitzka and Siva, 2007). Triacylglycerols can be extracted from these algae by osmotic cell lysis or solvent extraction (Wang et al., 2013). As a biofuel production platform, *Dunaliella* spp. are advantageous as they would not compete for arable land or freshwater for growth. *Dunaliella* spp. have been cultivated using a number of different nitrogen sources, including NH_4_^+^, NO_3_^-^, NO_2_^-^, NO, urea, histidine, glutamine, hypoxanthine, and allantoate (Goldman and Peavey, 1979; Latorella et al., 1981; Fabregas et al., 1989; Giordano et al., 1994; Giordano, 1997; Hellio and Le Gal, 1998; Nagase et al., 2001). Uptake of histidine in *Dunaliella tertiolecta* was demonstrated previously (Hellio and Le Gal 1998, 1999), but the mechanism of uptake, the identity of the transporter responsible, and any effects on metabolism remain unknown.

We determined the ability of four *Dunaliella* species to utilize 20 proteinogenic amino acids supplied individually as a sole nitrogen source. Only four amino acids [glutamine (Gln, Q), cysteine (Cys, C), histidine (His, H), and tryptophan (Trp, W)] recovered growth of *Dunaliella* from nitrogen starvation. Supplementation of these amino acids resulted in a set of unique metabolite profile changes. The mechanism by which *Dunaliella* spp. obtain nitrogen from these amino acids was also investigated. We found evidence for the uptake of histidine in one strain of *D. viridis*; however, it remains unknown how this amino acid is assimilated. In contrast, glutamine, cysteine, and tryptophan appear to oxidize in the presence of light, supplying NH_4_^+^ that is likely transported across the cell membrane by *Dunaliella* spp. and assimilated by the GS-GOGAT pathway.

## Materials and Methods

### Strains and Growth Conditions

The *Dunaliella* strains used in this study (*D. salina* CCAP 19/18, *D. viridis* dumsii, *D. tertiolecta* CCMP 364, *D. primolecta* UTEX LB1000) were obtained from the Culture Collection of Algae and Protozoa (CCAP^1^), National Center for Marine Algae and Microbiota (NCMA^2^), or UTEX^3^.

Strains were grown in 125 ml Erlenmeyer flasks containing modified Ben-Amotz media (mBA, pH 7.5) (Ben-Amotz et al., 1989; Srirangan et al., 2015) and maintained in exponential phase prior to inoculation of growth experiments. All cultures (except those used in uptake assays, see below) were grown at 21°C under continuous cool white fluorescent light with an intensity of 150 μmol photons m^-2^ s^-1^ at the culture surface.

### Nitrogen Source Growth Experiments

Nitrogen source growth experiments were carried out in batch culture using filter-sterilized mBA lacking nitrogen (mBA -N) medium in 4 ml volumes within 12-well polystyrene tissue culture plates. The cell density of *Dunaliella* cultures was quantified using a TC10 Automated Cell Counter (Bio-Rad). For each biological replicate, 8 × 10^7^ cells were harvested by centrifugation at 3,441 × *g* for 2 min. The supernatant was discarded and cultures were washed in mBA -N. These cell suspensions were again centrifuged, the supernatant was discarded, and cells were re-suspended in 1 ml of mBA -N. An inoculum of 4.0 × 10^6^ cells in a volume of 50 μl was used to seed each well containing media. For cultures of *D. salina*, only 2 × 10^6^ cells were used. Cells were mixed and the density of each well was recorded. Plates were then wrapped with parafilm and grown for 144 h under the conditions described above. At 0 and 144 h post-inoculation, cultures were mixed and the cell densities and diameter were determined. There were four biological replicates used for each *Dunaliella* strain.

### Urea Transporter and Enzyme Search

High affinity urea transport in plants is carried out by homologs of the yeast DUR3 urea transporter (Kojima et al., 2006). To find *Dunaliella* DUR3 homologs, yeast DUR3 (yeastgenome.org: YHL016C) and *Arabidopsis thaliana* AtDUR3 (TAIR: AT5G45380) protein sequences were used as BLAST queries against the *D. salina* CCAP 19/18 genome^4^ and against the assembled transcriptomes of *D. viridis* strain dumsii (Srirangan et al., 2015), *D. tertiolecta* CCMP 364, and *D. primolecta* UTEX 1000 (Matasci et al., 2014). Blast hits that also contained the conserved domain PF00474 were considered to be DUR3 homologs (Finn et al., 2016).

The metabolism of urea to ammonia in organisms can be accomplished via two different routes. The first consists of a nickel dependent urease producing ammonia and carbon dioxide. The second method uses urea carboxylase and allophanate hydrolase to convert urea to ammonium and bicarbonate through an allophanate intermediate (Fan et al., 2012). The urease protein sequence from *A. thaliana* (TAIR: AT1G67550) and the urea carboxylase (Phytozome_v11: Cre08.g360050.t1.1) and allophanate hydrolase (Phytozome_v11: Cre08.g360100.t1.2) protein sequences from *Chlamydomonas reinhardtii* (Merchant et al., 2007) were used as BLAST queries against the *D. salina* CCAP 19/18 genome^5^ and against the assembled transcriptomes of *D. viridis* strain dumsii (Srirangan et al., 2015), *D. tertiolecta* CCMP 364, and *D. primolecta* UTEX 1000 (Matasci et al., 2014).

### Metabolite Quantification

#### Chlorophyll

Culture samples of one ml were harvested by centrifugation for 10 min at 16,000 × *g*. The supernatant was removed and 625 μl of 100% EtOH was added to the remaining pellet. Samples were resuspended by vortexing and incubated at room temperature for 1 h. Samples were mixed every 15 min and then centrifuged for 10 min at 16,000 × *g*. The chlorophyll content was measured as absorption at 652 nm with 100% EtOH as a background control. The total chlorophyll amount (μg ml^-1^) was calculated as A_652_/36 (36 = extinction coefficient) (Arnon, 1949).

#### Neutral Lipid

##### Generation of coconut oil standards used in calibration curve

Absolute neutral lipid concentration was determined using coconut oil in mBA containing 0.01% Tween as a standard. Briefly, 10 mg of coconut oil was melted and added to 5 ml of mBA containing 0.2% Tween (2000 μg ml^-1^) and sonicated for 20 min using a Microson Ultrasonic Cell Disruptor set to 5 watts. The sonicated coconut oil solution was diluted in mBA containing 0.05% tween (500 μg ml^-1^) and sonicated for a further 20 min at 5 watts. Coconut oil standards of 5–100 μg ml^-1^ were prepared from freshly made 500 μg ml^-1^ coconut oil stocks by serial dilution using mBA containing 0.01% Tween.

##### Neutral lipid quantification

Neutral lipid accumulation was quantified using Nile Red (9-diethylamino-5H-benzo(α)phenoxazine-5-one; Sigma–Aldrich, United States) following the method of Elsey with modifications (Elsey et al., 2007). Freshly prepared 0.78 mM Nile red in acetone was added to a final concentration of 0.26 μM in each cell culture or coconut oil standard and mixed. Each resulting suspension was split into three 200 μl replicates in a polystyrene microplate and read with a microplate reader using fluorescence excitation of 485 nm and emission of 590 nm. Samples were incubated in darkness for approximately 15 min prior to reading. Sterile mBA, as well as mBA containing 0.01% tween were used as background controls for cell cultures and coconut oil standards, respectively.

#### Carbohydrate Quantification

Total carbohydrate concentration was determined by the Dubois method amended for use in a 96 well plate (Dubois et al., 1956). 0.5 ml of each cell culture was centrifuged for 10 min at 16,000 × *g*. The supernatant was removed and the cells were lysed in 0.5 ml of distilled H_2_O. Sucrose standards of 5 to 500 μg ml^-1^ were prepared in distilled H_2_O. Each standard or sample was split into triplicate 40 μl aliquots in clear polystyrene 96 well plates. Crystalline phenol in H_2_O was freshly made to a concentration of 5% w/v and 40 μl of was added to each sample and mixed. After 15 min, 200 μl of 95–98% sulphuric acid was added, and samples were immediately mixed 20 times by pipetting. Plates were cooled to room temperature data was quantified by measuring absorption at 490 nm using a microplate reader.

#### Soluble Protein

Soluble protein extraction and quantification was performed as previously described with modification (Srirangan et al., 2015). No β-mercaptoethanol was used for re-extraction.

### Quantification of Free NH_4_^+^ in Growth Medium

Free NH_4_^+^ in growth medium was quantified using the phenate method amended for use with mBA medium in a 96 well plate (Eppley et al., 1969). NH_4_^+^ standards of 5 to 2,000 μg ml^-1^ were made using NH_4_Cl in mBA -N. Each sample or NH_4_^+^ standard was added in triplicate 50 μl aliquots to 96 well microplate. To each aliquot, 40 μl of 20% (w/v) crystalline phenol in EtOH and 40 μl 0.5% (w/v) sodium nitroprusside in distilled H_2_0 were added and mixed. A 10 ml solution of 20% (w/v) sodium citrate and 2% NaOH in distilled H_2_O was combined with 3.5 ml of commercial bleach (7.25% sodium hypochlorite) and 70 μl of the resulting solution was added to each well and immediately mixed 10 times. Plates were developed for 1 h in darkness. Free NH_4_^+^ was quantified by measuring absorption at 630 nm.

### Uptake Assays

**FIGURE 2:**
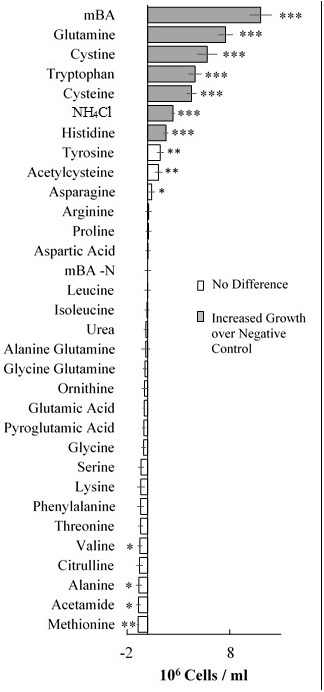
**Growth of *Dunaliella viridis* supplemented with nitrogen substrates.** The difference in mean cell density between cultures grown on different nitrogen sources and a nitrogen starvation control (mBA -N) was analyzed. Cultures were grown for 144 h on mBA -N containing 5 mM of each the above nutrients (0.5 mM used for NH_4_Cl). Error bars represent one standard deviation. Significant differences relative to the unsupplemented mBA -N control was assessed using a two-tailed paired Student’s *t*-Test at *p* ≤ 0.05 (^∗^), *p* ≤ 0.01 (^∗∗^), and *p* ≤ 0.001 (^∗∗∗^). Four biological replicates were used for analysis.

Amino acid uptake assays for *Dunaliella* spp. were amended from a yeast protocol (Su et al., 2004). Briefly, 50 ml *Dunaliella* cultures in mBA were maintained in a Conviron growth chamber (model number ATC60) under long-day condition (16 h light/8 h dark, 22°C/18°C, respectively) and 135 μmol photons m^-2^ s^-1^ in exponential phase prior to inoculation of growth medium containing different nitrogen sources. From each growth medium, either 5 × 10^7^ (**Figure 5A**) or 2 × 10^8^ (**Figure 5B**) cells were harvested by centrifugation and suspended in 1 ml of mBA -N. Each cell suspension was dispensed into 100 μl aliquots. Aliquots were maintained on ice until 10 μl of 1 M glucose was added to each suspension, after which cells were mixed and incubated for 5 min under cool white fluorescent light at 25°C (40 μmol photons m^-2^ s^-1^). Uptake solutions were prepared by mixing non-labeled amino acid (final concentration of 1 mM) with two microliters of the following labeled amino acids at mCi ml^-1^: L-cystine [3,3′-^14^C], L-histidine [2,5-^3^H], and L-glutamine [3,4-^3^H(N)]. Uptake solution was mixed with incubated cell aliquots, yielding a final specific activity of ∼91 nCi μl^-1^. A portion of each sample (50 μl) was taken at several time points and added to 5 ml of 1 M NaCl on a DHI Filtration Manifold (CAT# EQU-FM-10X20-SET) fitted with 24 mm glass filters (Whatman CAT# 1822-024). The solution was removed by application of vacuum, and the washing was repeated two more times with 5 ml 1 M NaCl. The filters containing the cells were loaded into scintillation vials, 500 μl of commercial bleach (5% sodium hypochlorite) was added to each scintillation vial. Radioactivity in each vial was measured using a Beckman-Coulter scintillation counter (LS6500 Multi-Purpose Scintillation Counter).

### NMR

#### Growth and Collection

Cultures used for NMR detection of metabolites were scaled into 3 L glass Erlenmeyer flasks containing 1 L of growth medium. After an initial period of scale up, cells used for inoculation were mixed once daily and maintained in exponential phase. To perform each growth experiment to be analyzed by NMR, 4.0 × 10^9^ cells were collected by centrifugation at 10,000 × *g* for 15 min. Supernatant was removed and cells were washed in mBA -N. Cells were then resuspended in 1 L of freshly made growth media supplemented with 5 mM Gln. Cell pellets and spent media was either immediately collected for extraction (*T* = 0) or grown in 3 L flasks for 24 or 48 h before collection. Cell pellets were collected by centrifugation at 10,000 × *g* for 15 min. The resulting spent media supernatant was immediately frozen at -80°C. The rest of the supernatant was discarded, and the remaining cell pellet was washed twice with 1 M NaCl. Wet cell pellets were immediately frozen at -80°C.

#### Metabolite Extraction

Metabolites were extracted from cell pellets with acetonitrile (Neumuller et al., 2013). Briefly, algal pellets were extracted with a 54% solution of acetonitrile in distilled H_2_O. Each extraction underwent 3 freeze-thaw cycles using liquid nitrogen and a 55°C water bath. Extractions were then centrifuged at 4,000 × *g* for 5 min. The resulting supernatant was collected in a 50 ml conical vial, and the remaining pellet was re-extracted with 4 ml of 60% acetonitrile. The re-extraction was centrifuged at 4,000 × *g* for 5 min and the resulting supernatant was combined with the prior supernatant. This combined supernatant was then frozen at -80°C.

#### ^1^H NMR Spectroscopy

The acetonitrile:water (3:2) extracted algae was dried on a speed-vac (Thermo-Fisher), and stored at -80°C until NMR analysis. The extracted powder was re-suspended in 600 μl of D_2_O containing 0.1 mM trimethylsilyl-2,2,3,3-tetradeuteropropioninc acid (TSP) and 1 mM formate and pipetted into a 5 mm NMR tube for subsequent high resolution NMR analysis. 1D one-pulse ^1^H-NMR spectra with water presaturation were acquired with 4 s recycle time using a 14.1T Varian INOVA (600MHz ^1^H frequency, Varian Instruments, Palo Alto, CA, United States) equipped with a 5 mm indirect detection probe.

#### NMR Spectral Analysis and Metabolite Identification

All NMR spectra were processed using ACD/1D NMR Manager software (version 12.0; Advanced Chemistry Development, Inc., Toronto, ON, Canada). Imported FIDs were zero-filled to 64,000 points, and an exponential line broadening of 0.3 Hz was applied prior to Fourier transformation. Spectra were phase and baseline corrected and referenced to the TSP peak at 0.00 ppm.

Metabolite identification was performed by comparing NMR spectral identifiers (chemical shifts, peak area ratios, peak multiplicity, coupling constants) were compared to metabolomic NMR databases [Human Metabolome Database^6^; University of Wisconsin^7^] and to known breakdown products of glutamate, such as pyroglutamate (Abraham and Podell, 1981). Metabolite quantification was determined using Chenomx software (version 5.1; Chenomx Inc., Edmonton, AB, Canada) as previously described (Dewar et al., 2010).

### UPLC-MS Determination of Amino Acid Content

The amino acid composition of *D. viridis* cells was determined using 8 ml of culture grown for 144 h. Samples were centrifuged for 10 min at 16,000 × *g*. Supernatant was removed and cells were lysed in 400 μl of distilled water. Cell lysates were stored at -20°C until derivatization. For total amino acid content analysis, 50 μl of frozen cell lysates were thawed and hydrolyzed using 1 ml vacuum hydrolysis tubes (Life Technologies) containing 400 μl of 6 N HCl with 0.02% β-mercaptoethanol. Hydrolysis was carried out under vacuum and 105°C for 20 h and terminated using 400 μl of 6 N NaOH.

Cell lysates and hydrolyzed cell lysates were separately derivatized with AccQ – Tag (Waters). Derivatized samples were run on a Waters UPLC system in reverse phase mode. Standard curves for each amino acid and ammonium were generated using amino acid standards from Sigma Aldrich (Catalog no.: AA-S-18) for a working range of 0 to 50 mM.

### Experimental Replication and Statistical Treatment

For all data presented, at least three biological replicates were used. Statistical analysis was performed in Microsoft Excel. A Student’s *t*-test was used for all comparisons between two means. Tukey’s HSD was used to establish significant groupings when performing multiple comparisons.

## Results

### *Dunaliella* spp. Are Susceptible to Ammonium Toxicity and Vary in the Ability to Use Urea

**FIGURE 3:**
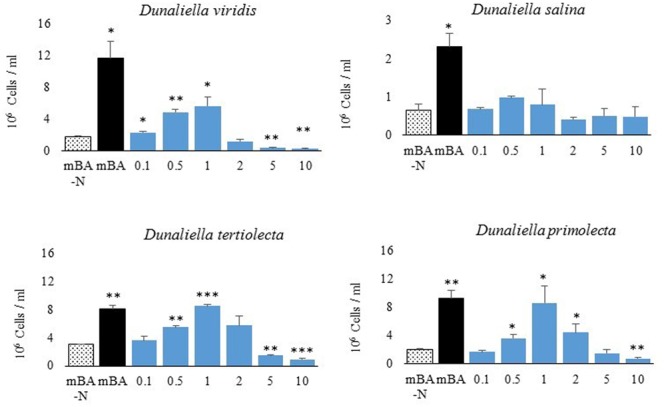
**Growth of *Dunaliella* spp. using NH_4_Cl as the sole nitrogen source.** Total cell density of cultures grown for 144 h on mBA-N containing NH_4_Cl at varied concentrations (in mM). Average cell density was measured from four biological replicates. Error bars represent one standard deviation. Significant growth relative to the un-supplemented mBA -N control was assessed using a two-tailed paired Student’s *t*-Test at *p* ≤ 0.05 (^∗^), *p* ≤ 0.01 (^∗∗^), and *p* ≤ 0.001 (^∗∗∗^). Four biological replicates were used for analysis.

A screen was conducted to determine whether various organic and inorganic nitrogen substrates could support growth of *D. viridis* (**Figure 2**). Two surprising results of this nutrient screen were that (i) urea did not support growth of *D. viridis* and that (ii) NH_4_^+^ in excess of 1 mM was generally inhibitory or toxic to all *Dunaliella* species tested (**Figure 3** and **Table 1**). Other *Dunaliella* species were able to grow to high density when supplemented with urea, and also possessed gene sequence encoding likely transporters and enzymes necessary for urea utilization (**Table 1**). In contrast, *D. viridis* lacked any available sequence that might code for a urea transporter, and also does not appear to possess a sufficient set of enzymes needed for urea conversion (**Table 1**). NH_4_^+^ above 2 mM was inhibitory regardless of the species tested, a situation previously observed only in *D. tertiolecta* (**Figure 3**; Chen et al., 2011). Ribonucleoside and nucleobase monomers were also evaluated as a nitrogen source, but did not rescue growth of nitrogen starved *D. viridis* (Supplementary Figure S1).

**Table 1:** Variation in the ability of *Dunaliella* spp. to use urea as a nitrogen source.

Organism	Growth	Number of Homologs Identified				Accessions	Reference
		Urea Transporter	Urease	Urea Carboxylase	Allophanate Hydrolase		
*D. viridis*		0	0	1	0	Locus_7748	(Srirangan et al. (2015))
*D. salina*	^∗∗^	3	0	1	1	Dusal.0292s00003, Dusal.0572s00011, Dusal.1401s00001, Dusal.0292s00001, Dusal.0292s00002	**^∗^**
*D. tertiolecta*	^∗∗∗^	1	0	1	1	ZDIZ-2004114, ZDIZ-2004115, ZDIZ-2051319	(Matasci et al. (2014))
*D. primolecta*	^∗∗∗^	1	0	1	1	WDWX-2002751, WDWX-2002752, WDWX-2045758	(Matasci et al. (2014))

*^∗^http://phytozome.jgi.doe.gov/. Growth denotes an observed increase in average final cell density of cultures grown with mBA –N containing 5 mM urea compared to the unsupplemented mBA –N control. Significant growth relative to an un-supplemented mBA -N control was assessed using a two-tailed paired Student’s *t***-**Test at *p* ≤ 0.05 (^∗^), *p* ≤ 0.01 (^∗∗^), and *p* ≤ 0.001 (^∗∗∗^). Three biological replicates were used for analysis. Putative homologs for Urea Transporter, Urease, Urea Carboxylase, and Allophanate Hydrolase were identified using BLAST (see Materials and Methods).*

### Dunaliella Cells Grow on Four Amino Acids as a Sole Nitrogen Source

**FIGURE 4:**
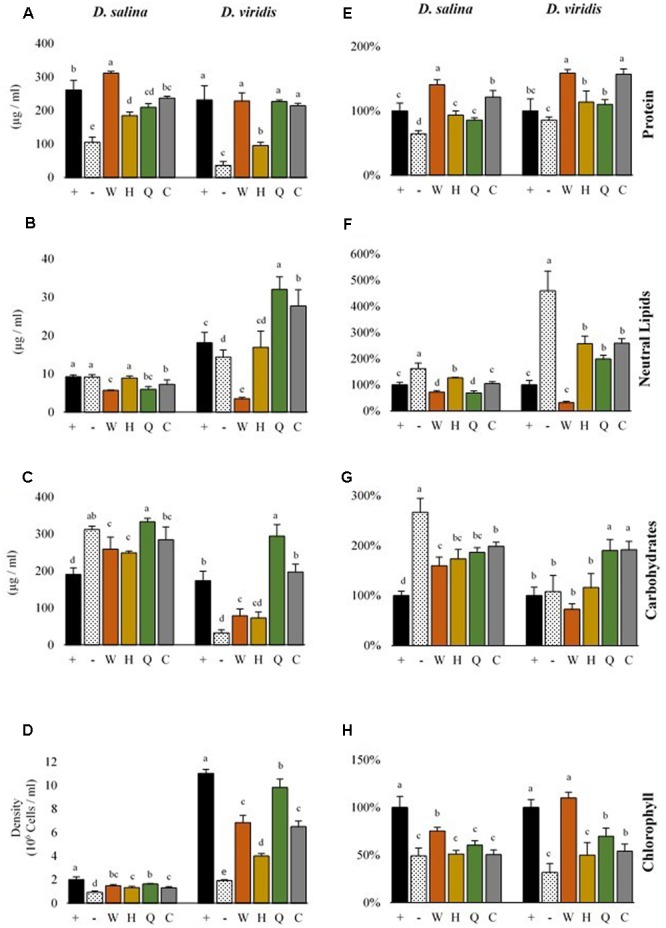
**Metabolite productivity and content of *D. salina* and *D. viridis.* Abbreviations: + (mBA), - (mBA -N), W (tryptophan), H (histidine), Q (glutamine), C (cysteine).** Volumetric productivity (left column) of soluble protein **(A)**, neutral lipids **(B)**, total carbohydrates **(C)**, and cell density **(D)** of cultures grown for 144 h. The cell content of soluble protein **(E)**, neutral lipids **(F)**, total carbohydrates **(G)**, and chlorophyll **(H)** is expressed relative to ${\mathrm{KNO}}_{3}^{–}$ supplemented cultures (mBA), which are used as a reference (100%). Error bars represent one standard deviation. Statistically significant population groupings are indicated (a,b,c,d,e; Tukey’s HSD; α ≤ 0.05). Four biological replicates were used for analysis.

Our screen revealed that compared to a nitrogen starvation control, cultures of *D. viridis* could reach high cell densities when supplemented with the amino acids Gln, His, Cys, and Trp (**Figures 2, 4** and **Table 2**). The other 16 proteinogenic amino acids either had no observable effect on growth, or caused a small decrease in final cell density. However, glycine was an exception under one condition. When glycine (5 mM) was mixed with KNO_3_ (5 mM) cultures had a final cell density and neutral lipid productivity nearly 50% greater than cultures grown with KNO_3_ alone (Supplementary Figure S2). This initial nitrogen source screen was expanded to include three other species of *Dunaliella (D. salina, D. tertiolecta, D. primolecta*). We found that all four species consistently reached higher cell densities than nitrogen starvation controls when supplied with Gln, His, Cys, Trp (**Table 2**). Just as for *D. viridis*, the other 16 proteinogenic amino acids had no observable effect on growth.

**Table 2:** Growth of four *Dunaliella* strains supplemented with Gln, Cys, His, and Trp.

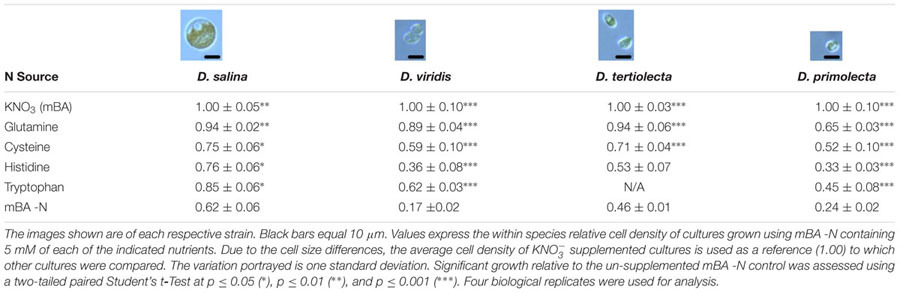

### The Availability of Nitrogen and Supplementation of Amino Acids Alters Metabolite Accumulation of *Dunaliella* spp. in a Species Specific Manner

There were differences in the level of metabolites accumulated by cells of each *Dunaliella* species, which was significant both when comparing between species and when comparing nitrogen supplementation treatments (**Figure 4**). While nitrogen starvation was generally associated with lower cell densities and decreased chlorophyll content, nitrogen-starved *D. salina* and *D. viridis* accumulated different levels of carbon storage metabolites and protein (**Figure 4**). Nitrogen-starved cultures of both *D. salina* and *D. viridis* accumulated higher amounts of neutral lipids on a cell basis, but *D. viridis* cells accumulated about five times more neutral lipids than ${\mathrm{KNO}}_{3}^{–}$ fed controls. Cells of *D. salina* accumulated about three times more carbohydrates under nitrogen starvation, while carbohydrate levels were unchanged in cells of *D. viridis* subjected to the same treatment (**Figure 4**). Cell protein abundance was significantly lower during nitrogen starvation in *D. salina*, but not in *D. viridis*.

Gln, His, Cys and Trp supplementation altered the metabolite profiles of *Dunaliella* in a species specific manner (**Figure 4**). While supplementation with each of four amino acids recovered the growth of *Dunaliella*, the color of these cultures was often different compared to KNO_3_ controls (Supplementary Figure S3). This color change was obvious even when comparing cultures of equivalent cell density. Consequently, each amino acid altered the metabolite profile of species in ways that were unique from both controls and other amino acids.

Gln supplementation yielded final cell densities and cell protein content that were nearly as high as a KNO_3_ control (**Figure 4**). However, for both *D. viridis* and *D. salina* the chlorophyll content in Gln supplemented cultures tended to be lower, and Gln supplemented cultures *D. viridis* were visibly yellow (Supplementary Figure S3). Compared to KNO_3_ controls, Gln supplemented cultures had almost double cellular carbohydrate content and consequently produced at least 50% more carbohydrates in total. Gln was also associated with a twofold higher level of cellular triacylglycerol production in *D. viridis* (**Figure 4**).

Although cultures supplemented with Cys did not grow as well as those supplemented with Gln, they accumulated similar levels of metabolites (**Figure 4**). Like Gln cultures, Cys supplemented cultures were also visibly yellow (Supplementary Figure S3). Neutral lipids, carbohydrates, and chlorophyll were present at equivalent levels in both treatments in *D. viridis*. However, Cys supplementation produced higher cell protein content than Gln or KNO_3_ cultures (**Figure 4**).

The growth rate of *Dunaliella* cultures supplemented with His remained low for all tested species (**Table 2**). Protein levels of His-fed cultures were only marginally higher than nitrogen-starved controls (**Figure 4**). As was the case for Gln and Cys supplementation, His-supplemented cultures had low chlorophyll content, giving rise to a yellowish appearance (Supplementary Figure S3).

Trp added to growth media produced a vibrant orange color after incubation for several days and exposure to light (Supplementary Figure S3). This effect also occurred in sterile media. Trp-supplemented cultures were often contaminated with bacteria [data not shown], which was never seen for the other amino acids. Data could only be obtained for cultures that had undergone a rigorous selection procedure to isolate cultures free of contaminating bacteria (Supplemental Methods). Cultures supplemented with Trp were unique in that both protein and chlorophyll levels tended to be highest in these treatments (**Figure 4**). While Trp supplementation marginally increased the carbohydrate content of *D. salina*, it reduced the neutral lipid content of *D. viridis* cells to 31% of KNO_3_ fed cells. Trp fed cultures produced one fifth the volume of neutral lipids compared to KNO_3_ controls. This effect was not observed in other species, but occurred even if *D. viridis* was simultaneously cultured with KNO_3_ and Trp (Supplementary Figure S2).

### Evidence of Amino Acid Assimilation Routes

The mechanism by which *Dunaliella* spp. are able to obtain nitrogen from the amino acids His, Gln, Trp, and Cys was investigated using the ecotype *D. viridis* as a model. Routes supported by prior literature include transport of amino acids across the cell membrane and recovery of NH_4_^+^ separated from amino acids either by abiotic oxidation chemistry or the activity of a deaminating enzyme (Kirk and Kirk, 1978a; Abraham and Podell, 1981; Palenik and Morel, 1990, 1991; Gracanin et al., 2009).

**FIGURE 5:**
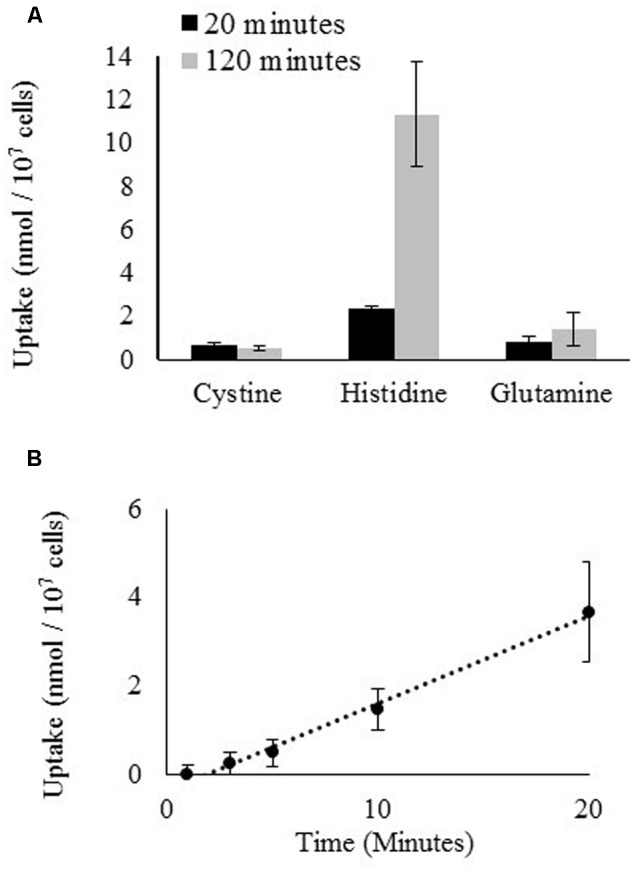
**Uptake of radiolabeled amino acids by *D. viridis.* (A)** Accumulation of L-cystine [3,3′-^14^C], L-histidine [2,5-^3^H], and L-glutamine [3,4-^3^H(N)] in cells of *D. viridis* after 20 and 120 min when supplied with 1 mM amino acids. **(B)** Time course kinetics of L-histidine [2,5-^3^H] uptake at 1 mM in mBA -N. Means ± Standard error of three biological replicates are shown.

The uptake of radiolabeled Gln, cystine, and His by *D. viridis* was measured over a period of 120 min (**Figure 5A**). Cystine was used as a proxy for Cys because of the relative instability of Cys, which rapidly oxidizes to cystine in aqueous solution (Kendall and Nord, 1926). No uptake of cystine or Gln was detected, only His was significantly taken up. This was true even if the culture used for the uptake assay was nitrogen-starved for 72 h prior to the assay (Data not shown). The uptake for radiolabeled His was approximately linear over a period of 20 min (**Figure 5B**).

**FIGURE 6:**
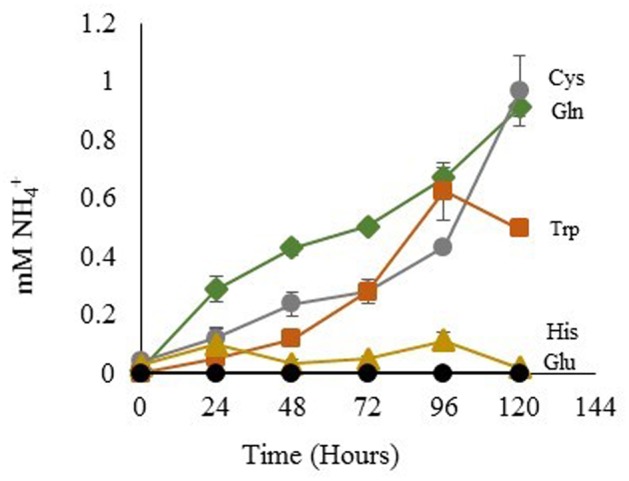
**NH_4_^+^ Released from amino acids in sterile media.** Glutamic Acid ●; tryptophan 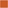; histidine 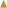; glutamine 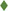; cysteine 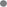. Means ± Standard Deviation of three independent replicates are shown.

The degree of NH_4_^+^ production by sterile His, Gln, Glu, Cys, and Trp-containing media was assessed using the same light and temperature conditions that are used for growth of *Dunaliella* cultures. Gln, Cys, and Trp media generated increasing NH_4_^+^ concentrations over time (**Figure 6**). In contrast, His-containing media gave an initially high signal, but the apparent NH_4_^+^ concentration of this media did not significantly change over time. Glu was used as a control and did not produce any detectable amount of free NH_4_^+^.

**FIGURE 7:**
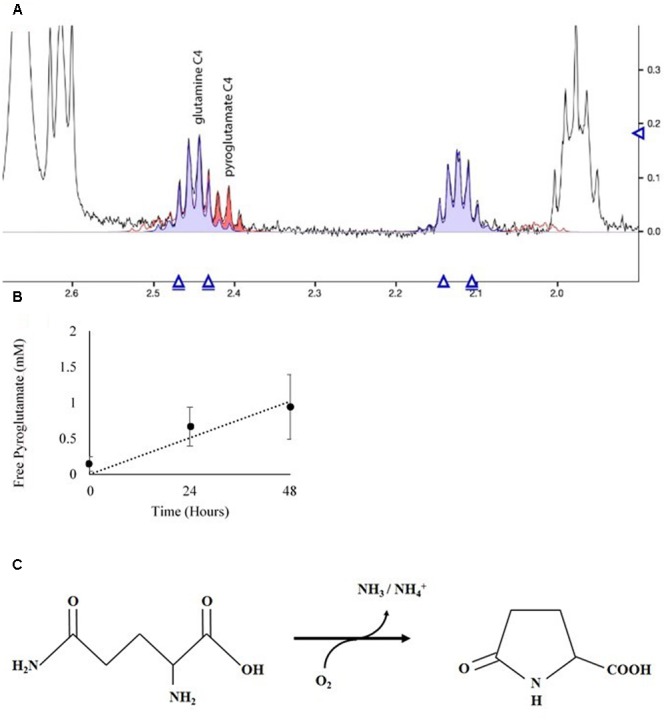
**Spectral analysis of spent growth medium containing glutamine. (A)** Portion of the NMR spectrum delineating the C4 position of glutamine and its cyclic breakdown compound, pyroglutamate. **(B)** Time course of pyroglutamate formation in spent media. Means ± Standard Deviation of three independent replicates are shown. **(C)** Oxidation reaction diagram of glutamine in aqueous solution under oxidizing conditions.

The NMR spectra of media was used to ascertain the fate of Gln in culture. Spent media from *D. viridis* cultivated with mBA -N containing Gln revealed that peaks corresponding to Gln were present at all time points, and that the absolute value of these peaks did not change significantly (**Figure 7**). However, a set of peaks corresponding to pyroglutamate appear prominently after 24 h, and the strength of this signal increased at 48 h (**Figure 7**). The estimated rate of pyroglutamate formation in this experiment was considerably higher than that obtained for the abiotic production of NH_4_^+^ from Gln-containing media (**Figures 6, 7**).

### Amino Acid Content of Dunaliella viridis

The amino acid content of *D. viridis* was obtained in order to ascertain what proportion of the biomass could be recycled using Gln, His, Trp, and Cys as fertilizer supplementation. There was little variation in the overall amino acid content of *D. viridis* even between nitrogen-depleted and ${\mathrm{KNO}}_{3}^{–}$ supplemented cultures (**Table 3**). The sum of relative Gln, His, Trp, and Cys fractions was at most 16% of the total amino acid fraction.

**Table 3:** Amino acid content of *D. viridis.*

Amino Acid	mBA	mBA –N + 0.5 mM NH_4_^+^	mBA -N
His	**1.30% ± 0.04**	**0.46% ± 0.21**	**1.61% ± 0.06**
Ser^1^	8.03% ± 0.43	9.83% ± 1.33	8.49% ± 0.51
Arg	4.20% ± 0.18	3.99% ± 0.59	4.09% ± 0.16
Gly	9.72% ± 0.72	8.69% ± 1.19	8.70% ± 0.31
Asp/Asn^2^	10.26% ± 0.80	10.07% ± 1.41	9.60% ± 0.39
Gln/Glu^2^	**13.91% ± 0.92**	**13.68% ± 1.97**	**13.06% ± 0.56**
Thr	4.73% ± 0.28	4.89% ± 0.72	5.06% ± 0.18
Ala	12.38% ± 0.95	12.31% ± 1.45	12.16% ± 0.41
Pro	5.30% ± 0.44	4.11% ± 0.44	5.61% ± 0.18
Cys	**0.52% ± 0.18**	**0.22% ± 0.07**	**0.29% ± 0.02**
Lys	6.22% ± 0.36	6.07% ± 0.79	6.16% ± 0.23
Tyr	3.51% ± 0.16	4.57% ± 0.51	3.45% ± 0.13
Met	1.77% ± 0.18	1.99% ± 0.25	1.94% ± 0.07
Val	4.66% ± 0.14	5.22% ± 1.16	5.21% ± 0.21
Ile	2.75% ± 0.09	2.56% ± 1.02	3.10% ± 0.11
Leu	7.36% ± 0.46	7.81% ± 1.27	7.96% ± 0.30
Phe	3.00% ± 0.18	3.22% ± 0.52	3.30% ± 0.11
Trp	**0.39% ± 0.02**	**0.30% ± 0.01**	**0.20% ± 0.06**
**Sum of His, Gln/Glu, Cys, Trp**	16.11% ± 1.16	14.67% ± 2.27	15.16% ± 0.69

*^1^A major component of mBA is tris, which eluted at a retention time close to serine, preventing accurate quantification. ^2^This data does not differentiate between glutamine and glutamic acid, or between asparagine and aspartic acid due to conversion during hydrolysis. Bolded values in this table highlight the amino acids most relevant to the text of this study.*

## Discussion

We surveyed the potential of using amino acids and other organic and inorganic nitrogen sources to support growth of four species from the genus *Dunaliella*. *D.* viridis was the only species that uniquely did not use urea as a nitrogen source, likely because the dumsii ecotype used in this study does not possess a urea transporter or an allophanate hydrolase (**Table 1**). In contrast to prior reports, we found that *Dunaliella* spp. are susceptible to NH_4_^+^ toxicity above 1 mM (**Figure 3**).

**FIGURE 8:**
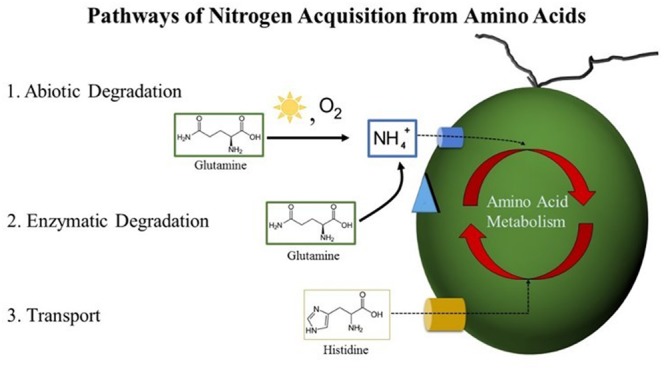
**Pathways of nitrogen acquisition from amino acids by *Dunaliella* spp.** Postulated routes by which nitrogen from amino acids could be acquired by *Dunaliella* spp. from the growth medium. (1) Abiotic degradation resulting in the production of NH_4_^+^ can occur via nucleophilic attack of primary amines by atmospheric oxygen catalyzed by the presence of heat and light. Released NH_4_^+^ can cross the cell membrane via a transport protein into the cell where it is assimilated via the GS-GOGAT pathway. (2) Enzymatic degradation of amino acids could occur if a *Dunaliella* species possesses an enzyme similar to the LAO1 protein of *C. reinhardtii* or another similar protein. This pathway would in effect release NH_4_^+^, which would be assimilated as in pathway 1. (3) Transport of amino acids across the cell membrane is mediated by a transporter. Once inside the cell, amino acids can be enzymatically degraded either by conversion into other amino acids via transamination or by the release and subsequent assimilation of NH_4_^+^.

Four amino acids supported growth of *Dunaliella* spp.: Gln, Cys, His, and Trp. Based on their existence in other algae, we investigated three potential routes by which *Dunaliella* spp. could acquire nitrogen from Gln, Cys, His, and Trp (**Figure 8**). No evidence was obtained that supports the existence of an extracellular amino acid oxidase in *Dunaliella* spp., and only His showed evidence of uptake across the cell membrane. Gln, Cys, and Trp release NH_4_^+^ via an abiotic process that *Dunaliella* spp. cells probably transport and utilize as nitrogen source.

### Dunaliella viridis Lacks the Necessary Genes for both Urea Transporters and Allophanate Hydrolase

*Dunaliella viridis* could not use urea as a nitrogen source (**Figure 2** and **Table 1**). This is in contrast to other *Dunaliella* spp., which recover growth when supplemented with urea (**Table 1**; Hellio and Le Gal 1998, 1999). The use of urea as a nitrogen source requires at least two components: urea uptake via a membrane transport protein and subsequent enzymatic degradation into HCO_3_^-^/ CO_2_ and NH_4_^+^ (Fan et al., 2012). NH_4_^+^ released by these two activities can then be assimilated via the GS-GOGAT pathway. We therefore probed the genomes of each *Dunaliella* species for the presence of sequence encoding putative urea transporters, urease, urea carboxylase, and allophanate hydrolase. No predicted proteins similar to urea transporters could be detected in the available genomic resources for *D. viridis* (**Table 1**). However, sequences putatively coding for these transporters could be identified in the other species of *Dunaliella*. The surveyed *Dunaliella* species do not contain any coding sequences for urease (**Table 1**). *D. salina, D. tertiolecta*, and *D. primolecta* contain sequences for urea carboxylase and allophanate hydrolase, while *D. viridis* only contains a sequence for urea carboxylase (**Table 1**). *D. salina, D. tertiolecta*, and *D. primolecta* grow when supplemented with urea as a sole nitrogen source and possess three necessary proteins that enable urea acquisition and metabolism. In contrast, this particular ecotype of *D. viridis* likely cannot utilize urea because it does not possess a transporter to carry urea across the cell membrane for subsequent assimilation. Even if *D. viridis* had an unknown protein capable of urea transport, it could convert urea to allophanate, but could not complete the conversion to ammonium and bicarbonate unless it possesses an unknown or alternate enzyme rescuing the activity of allophanate hydrolase.

One caveat is that the genomic resources available for *D. viridis* are derived entirely from RNA sequencing. Urea transporter and allophanate hydrolase mRNAs may not have been captured in the samples used for sequencing. We probed for the presence of urea transporter and allophanate hydrolase genes in the genomic DNA of *D. viridis* using consensus primers derived from the sequence of other *Dunaliella* spp. No putative urea transporter or allophanate hydrolase genes were amplified from *D. viridis* genomic DNA using *D. salina, D*. *tertiolecta*, and *D*. *primolecta* genomic DNA as positive controls (Data not shown). It is therefore highly unlikely that *D. viridis* possesses sequence coding for these two genes.

Urea carboxylase, allophanate hydrolase, and a urea transporter are grouped sequentially together within the *D. salina* genome^8^. This gene arrangement is also reflected on chromosome 8 of the *C. reinhardtii* genome (Merchant et al., 2007) and scaffold 3 of *Volvox carteri* (Prochnik et al., 2010), both species within the Chlamydomonadales order along with *Dunaliella*. If this syntenic relationship is conserved within the Chlamydomonadales order, this block of genes may have been deleted in *D. viridis*.

### NH_4_^+^ Toxicity Varies with Experimental Methodology

Although NH_4_^+^ is a useful source of nitrogen for photosynthetic organisms, it is also toxic at high concentrations. NH_4_^+^ toxicity is due to the decoupling effect of NH_4_^+^/ NH_3_ equilibrium, which can disturb membrane proton and charge gradients (Krogmann et al., 1959). Surprisingly, NH_4_^+^ supplementation of the *Dunaliella* cultures used in this study is toxic at concentrations less than previously reported to be suitable (5 mM) (**Figure 3**; Giordano et al., 1994; Giordano, 1997). Instead, we found growth maxima for some *Dunaliella* spp. at 1 mM NH_4_Cl, with higher concentrations causing inhibition of growth or cell death. It is therefore necessary to understand what factors contribute to the ability of some *Dunaliella* spp. to tolerate this nutrient better than others. However, there are several key differences in the methods used to cultivate *Dunaliella* spp. between our study and those prior that may explain this discrepancy.

Acclimation of *Dunaliella* spp. to the presence of NH_4_^+^ may affect the ability of these organisms to resist gradient decoupling effects. *Dunaliella* spp. have a long life cycle (0.47 – 1.22 divisions per 24 h) (Oren, 2005), and therefore may require substantial time to shift genetic and protein machinery when first exposed to NH_4_^+^. Prior studies involving higher amounts of NH_4_^+^ were materially different than ours in that the *Dunaliella* cultures used were acclimated to NH_4_Cl as a sole nitrogen source prior to the start of experimentation (Giordano et al., 1994; Giordano, 1997). In contrast, the cultures used in our study were grown using KNO_3_ as a sole nitrogen source prior to experimentation.

Another important factor affecting NH_4_^+^ toxicity is the amount of headspace and airflow in the container used for algal cultivation. NH_4_^+^/ NH_3_ equilibrium increasingly favors the production of NH_3_, a volatile gas, under more basic conditions. The algal cultivation media used in this study begins neutral (pH 7.5), but becomes basic (pH 10) after 72 h. It is likely that as *Dunaliella* growth experiments progress, NH_3_ leaves the liquid phase of the cultures and lowers the effective NH_4_^+^ concentration in the media. Additionally, this process should proceed to an equilibrium given a limited space for gas expansion. As NH_3_ volatilizes out of liquid over time and occupies the headspace of its container, NH_4_^+^/ NH_3_ equilibrium will be further driven toward NH_3_ production, likely lowering the effective NH_4_^+^ concentration. This means that the headspace of its cultivation vessel likely has a large effect on the NH_4_^+^/ NH_3_ equilibrium and therefore the effective NH_4_^+^ concentration. Compared to other studies using NH_4_^+^ supplementation in *Dunaliella* growth experiments, the 12 well tissue culture plates used in this study have less headspace. It is therefore reasonable to assume that the reason our cultures were more susceptible to NH_4_^+^ toxicity was that they were subjected to higher effective levels of NH_4_^+^.

### Gln, Cys, and Trp Supply Nitrogen via the Release and Assimilation of NH_4_^+^

Gln and Cys support growth of *Dunaliella* spp., but also increase the amount of carbon storage metabolites of cells compared to KNO_3_ fertilizer controls. It is unlikely to be the case that *Dunaliella* spp. can access or assimilate the carbon from Gln and Cys, as we found no evidence that these amino acids cross the cell membrane. Therefore, one likely reason for increased carbon storage is that Gln and Cys actually supply NH_4_^+^ via their oxidative degradation, which is a more efficient source of nitrogen than KNO_3_. Cells must convert KNO_3_ to NH_4_^+^ before nitrogen can be fixed to Glu via the GS-GOGAT cycle. This conversion process requires eight electrons, which are ultimately derived by light capture during photosynthesis. When NH_4_^+^ is assimilated, the eight electrons that would have otherwise been needed for nitrate assimilation can be either utilized elsewhere, i.e., for carbon fixation, or the cell can reduce its rate of electron capture from water oxidation by lowering light harvesting activity and chlorophyll content. This is consistent with the observed levels of chlorophyll in Gln and Cys-supplemented cultures of both *D. salina* and *D. viridis*, which possessed significantly lower cellular chlorophyll levels than KNO_3_ fertilizer controls (**Figure 4** and Supplementary Figure S3). Surprisingly, Gln and Cys-supplemented cultures produced higher cell concentrations of carbohydrates and neutral lipids while retaining similar amounts of protein compared to KNO_3_ controls. NH_4_^+^ utilization is consistent with these metabolic effects, as this scenario allows that less electron capture could exist simultaneously with increased carbon capture.

Although Trp releases NH_4_^+^ in solution like Cys and Gln, Trp supplementation resulted in uniquely elevated chlorophyll and protein levels as well as diminished neutral lipid content in *D. viridis*. These cultures were also commonly susceptible to contamination by bacteria, unlike other nutrient regimes we tested. Trp is also unique because it degrades into a variety of products due to light catalyzed oxidation of the pyrrole ring (Gracanin et al., 2009). This process is fast enough to be visually apparent, as cultures were visibly orange after as little as 24 h. Therefore, in addition to NH_4_^+^, there remains the possibility that one of many Trp degradation products or Trp itself is sensed or acquired by *Dunaliella* spp.

### Extracellular Enzymatic Release of NH_4_^+^ from Amino Acids Unlikely to Occur in *Dunaliella* spp.

Extracellular deamination of amino acids by an enzyme is known to occur in microalgae (Munoz-Blanco et al., 1990; Palenik and Morel, 1990, 1991; Piedras et al., 1992; Vallon et al., 1993). Although there was no prior evidence for their existence in *Dunaliella* spp., we did not rule out the possibility that at least one of those enzymes was present and active. *Dunaliella* spp. are in the same Order as *C. reinhardtii*, which possesses a non-specific L-amino acid oxidase (LAO1) located in the periplasmic space that deaminates a wide range of amino acids, releasing NH_4_^+^ and corresponding keto-acids (Munoz-Blanco et al., 1990; Piedras et al., 1992; Vallon et al., 1993). We probed the genomes of four species of *Dunaliella* and could not find any protein-coding genes of significant similarity to LAO1 (Data not shown). The fact that *Dunaliella* cells grew only in the presence of amino acids subject to abiotic deamination does not support the hypothesis of the existence of an amino acid oxidase. Additionally, our NMR data reveal that cultures of glutamine-fed *D. viridis* form pyroglutamate outside the cell (**Figure 7**). If activity analogous to LAO1 existed we expect to instead find a signal corresponding to 2-keto-glutaramic acid.

### Histidine Transport and Use Is Inefficient in *Dunaliella* spp.

Uptake of His has now been demonstrated in two species of *Dunaliella* and appears to support growth in each of the species tested (**Table 2** and **Figure 5**; Hellio and Le Gal 1998, 1999). We therefore speculate that His uptake and utilization may be a general feature of this genus. Although His is transported across the cell membrane, His supplementation conferred little improvement in growth or accumulation of major carbon or nitrogen metabolites over cultures that were starved for nitrogen. This is surprising, as transported His should functionally supply nitrogen and carbon via conversion by histidine ammonia lyase. However, the kinetics of His uptake, the identity of the transporting protein, and the mechanism of transport are unknown. This situation is intriguing because a parallel situation exists in the model alga *C. reinhardtii*. *C. reinhardtii* efficiently transports only one amino acid, arginine, which like histidine is positively charged, and the identity and mechanism of transport and assimilation of arginine are unknown. This similarity might yield an evolutionary insight if the respective transport mechanisms could be identified.

It is unlikely that His is able to supply nitrogen via enzymatic or abiotic degradation outside the cell, because light catalyzed degradation of His does not yield free NH_4_^+^, but aspartate and urea (Tomita et al., 1969). However, aspartate and urea do not support growth of *D. viridis.* Therefore, only His itself could supply nitrogen to cells of *D. viridis*. Additionally, His supplementation only marginally improved growth even in cultures that can use urea as a nitrogen source.

## Conclusion

Gln, Cys, His, and Trp supports growth of *Dunaliella* spp. but are currently an insufficient means of nitrogen recycling from spent biomass. These four amino acids constitute at most 16% of the proteinaceous amino acid pool (**Table 3**) in *D. viridis*, and this value is likely inflated as our data does not distinguish values for Glu from Gln. Furthermore, the use of His only marginally improves growth and the use of Trp presents a contamination risk.

The fact that the same four proteinogenic amino acids consistently enabled growth of *Dunaliella* regardless of species is knowledge transferable to other work in the development of algal bioproducts. The release of NH_4_^+^ from Gln, Trp, and Cys explains part of this consistency, as all *Dunaliella*, and indeed most photosynthetic microorganisms can use NH_4_^+^ as a nitrogen source. Consequently, we predict that any alga which can use NH_4_^+^ as a nitrogen source should also grow when supplemented with Gln, Trp, or Cys. Another implication is that like anaerobic digestion and hydrothermal liquefaction, the *in vitro* digestion scheme forming the basis of this work (**Figure 1**) must consider the abundance and impact of ammonium generated from abiotic amino acid degradation. Given this state, it would appear to be simpler to digest *Dunaliella* biomass using conventional means and recycle nitrogen as NH_4_^+^. However, if *Dunaliella* or another alga could use whole amino acids as both a carbon and nitrogen source the impact of NH_4_^+^ would be reduced.

We had assumed that there would be variation in the use of amino acids between the *Dunaliella* species as these species are from different geographic areas of the world. However, the limited use of amino acids is not unique to *Dunaliella*, or to marine or halophilic microalgae generally. This yields an opportunity for genetic optimization, where transgenic *Dunaliella* or other algae expressing recombinant amino acid transporters or extracellular deaminating enzymes may allow greater usage of amino acid nitrogen.

Amino acid proportion of *D. viridis* cultivated for 144 h determined by UPLC-MS. Values above are expressed as the relative percentage of the total amino acid content of cultures. These values include both free amino acids and hydrolyzed total protein. The variation portrayed is one standard deviation. Values are derived from three biological replicates.

## Author Contributions

CM, JM, GP, and HS were involved in the conception and design of this project. CM, JD, SJ, CZ, DY, NK, and AT were involved in collection and assembly of data. CM, CZ, AT, JM, GP, and HS were involved in the analysis and interpretation of the data. All authors were involved in the drafting of this article. CM, JD, JM, GP, and HS were responsible for critical revision of the article for important intellectual content. All authors were involved in final approval of the article.

## Conflict of Interest Statement

The authors declare that the research was conducted in the absence of any commercial or financial relationships that could be construed as a potential conflict of interest.

## Footnotes

1 http://www.ccap.ac.uk/

2 https://ncma.bigelow.org/

3 https://utex.org/

4 http://phytozome.jgi.doe.gov/

5 http://phytozome.jgi.doe.gov/

6 http://www.hmdb.ca

7 http://www.bmrb.wisc.edu/metabolomics/

8 http://phytozome.jgi.doe.gov/
