# Supplementary material for: Amino Acids Are an Ineffective Fertilizer for Dunaliella spp. Growth
Source: Front Plant Sci. 2017 May 26;8:847. doi: 10.3389/fpls.2017.00847 (PMC5445130; doi:10.3389/fpls.2017.00847)
Supplement: Supplementary file 1 [file Data_Sheet_1.DOCX]

# **S1: Growth of *D.* *viridis* dumsii on Ribonucleosides and Nucleobases as sole N-source.**

The total cell density of cultures grown for 72 hours in mBA -N containing each of the above metabolites at the indicated concentrations. Average cell density was measured from four biological replicates. Error bars represent one standard deviation. Significant growth relative to the mBA – N control was assessed using a two tailed paired Student’s t**-**Test at p ≤ 0.05 (*), p ≤ 0.01 (**), and p ≤ 0.001 (***).

# **S2: Productivity of *Dunaliella viridis* Supplemented with Amino Acids and KNO_3_**

The difference in mean absolute cell density and volumetric lipid productivity between cultures and a KNO_3_ control (mBA) is shown. Cultures were grown for 144 hours on mBA media containing 5 mM of both KNO_3_ and each the above amino acids. Error bars represent one standard deviation. Significant differences relative to the mBA control were assessed using a two tailed paired Student’s t**-**Test at p ≤ 0.05 (*), p ≤ 0.01 (**), and p ≤ 0.001 (***). Values are derived from three biological replicates.

His

Cys

Gln

Trp

mBA

mBA -N


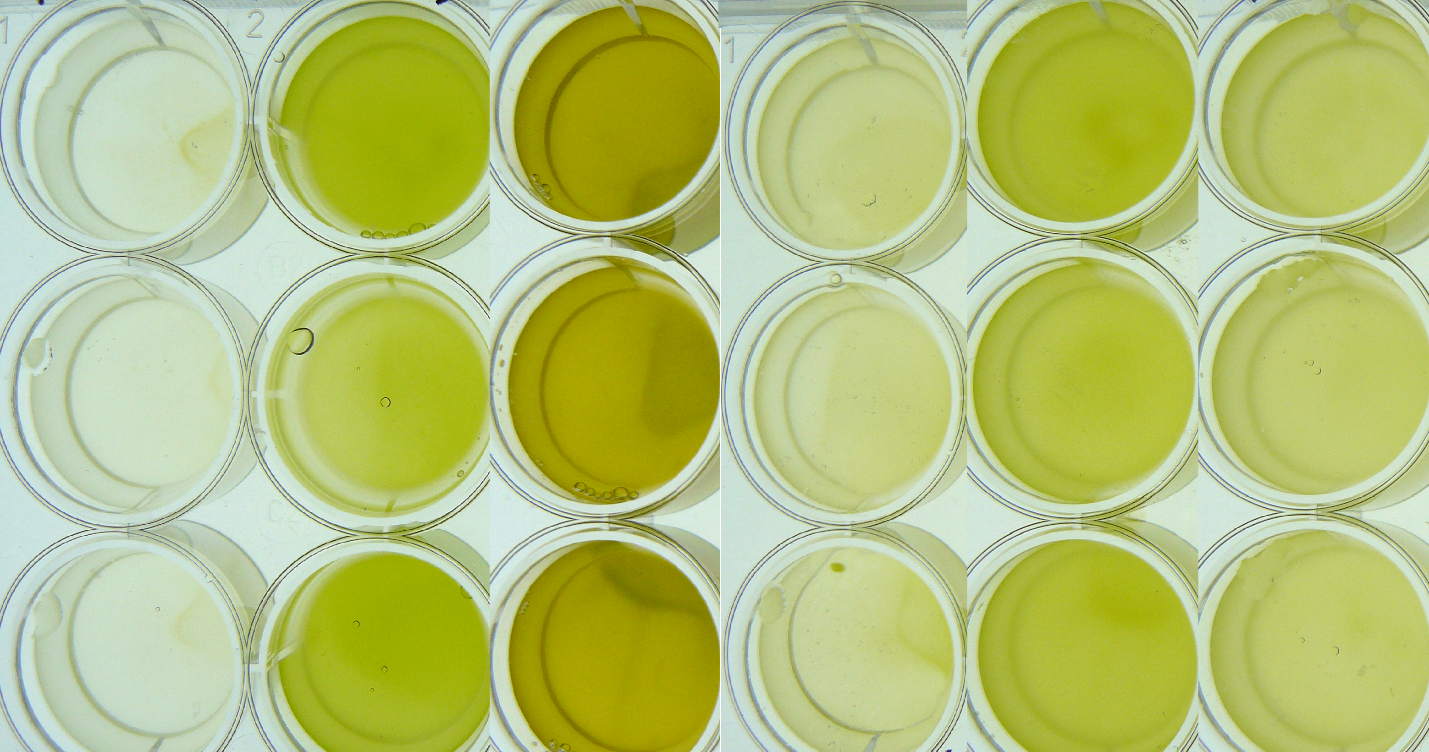


# **S3: White light Photographs of *Dunaliella viridis* Cultures**

Abbreviations: Trp, Tryptophan; His, Histidine; Gln, Glutamine; Cys, Cysteine. Light photographs of 4 ml *D. viridis* dumsii cultures grown for 144 hours using mBA –N containing 5 mM of the above amino acids.
