# Supplementary material for: Amino Acids Are an Ineffective Fertilizer for Dunaliella spp. Growth
Source: Front Plant Sci. 2017 May 26;8:847. doi: 10.3389/fpls.2017.00847 (PMC5445130; doi:10.3389/fpls.2017.00847)
Supplement: Supplementary file 2 [file Data_Sheet_2.DOCX]

## Supplemental Methods

**Supplemental Methods 1: Cleaning Procedure to Isolate Pure Culture**

The *Dunaliella* strains used in this study (*Dunaliella viridis* dumsii, *D. tertiolecta* CCMP 364, *D. primolecta* LB 1000, *D. salina* 19/18) were isolated as pure culture using a modification of a protocol obtained from UTEX (https://utex.org/). Briefly, 50 ml cultures were maintained under autotropic growth conditions (Methods 2.1) and pelleted at 300 xg for 3 minutes. Pellets were suspended in 1 ml of mBA, transferred to a new tube, and brought to 50 ml with mBA. The resulting cultures were sonicated for 10 seconds. The centrifugation through sonication steps were repeated a total of seven times. 100 µg/ml each of carbenicillin, kanamycin, rifampicin, and spectinomycin were added to the resulting 50 ml cultures, which were then grown for 72 hours under autotrophic conditions. Recovering cultures were serially diluted to 100,000 cells per ml, and 200 µl of the resulting dilution was plated onto mBA 1 % phytoblend agar plates containing 100 µg/ml each of carbenicillin, kanamycin, rifampicin, and spectinomycin. These plates were grown under autotrophic conditions for at least two weeks, and the resulting individual colonies were transferred to new antibiotic-containing plates. Individual colonies on these subsequent plates were used to inoculate pure cultures. The presence or absence of contamination was established by the use of PCR targeting 16s rRNA.
